# 1-Deoxynojirimycin in Mulberry (*Morus indica L.*) Leaves Ameliorates Stable Angina Pectoris in Patients With Coronary Heart Disease by Improving Antioxidant and Anti-inflammatory Capacities

**DOI:** 10.3389/fphar.2019.00569

**Published:** 2019-05-21

**Authors:** Yan Ma, Wei Lv, Yan Gu, Shui Yu

**Affiliations:** ^1^Department of Cardiovascular, The First Hospital of Jilin University, Changchun, China; ^2^Department of Cadre Ward, Seven Therapy Area, The First Hospital of Jilin University, Changchun, China

**Keywords:** 1-deoxynojirimycin, mulberry leaves, coronary heart disease, angina pectoris, blood stasis syndrome

## Abstract

**Objective:** Stable angina pectoris (SAP) in patients with coronary heart disease (CHD) and blood stasis syndrome (BSS) is a potentially serious threat to public health. NF-κB signaling is associated with angina pectoris. 1-Deoxynojirimycin (DNJ), which is a unique polyhydroxy alkaloid, is the main active component in mulberry (*Morus indica L.*) leaves and may exhibit protective properties in the prevention of SAP in patients with CHD by affecting the NF-κB pathway.

**Methods:** DNJ was purified from mulberry leaves by using a pretreated cation exchange chromatography column. A total of 144 SAP patients were randomly and evenly divided into experimental (DNJ treatment) and control (conventional treatment) groups. Echocardiography and ascending aortic elasticity were evaluated. The changes in inflammatory, oxidative, and antioxidant factors, including C-reactive protein (CRP), interleukin-6 (IL-6), tumor necrosis factor-α (TNF-α), superoxide dismutase (SOD), and malondialdehyde (MDA), were measured before and after a 4-week treatment. Self-Rating Anxiety Scale (SAS) and Hamilton Depression Scale (HAMD) scores were compared between the two groups. The improvement in SAP score, associated symptoms, and BSS was also investigated. The levels of IkB kinase (IKK), nuclear factor-kappa B (NF-κB), and inhibitor of kappa B α (IkBα) were measured by Western blot.

**Results:** After the 4-week treatment, DNJ increased left ventricular ejection fraction and reduced left ventricular mass index, aortic distensibility, and atherosclerosis index (*p* < 0.05). DNJ intervention increased angina-free walking distance (*p* < 0.05). DNJ significantly reduced the levels of hs-CRP, IL-6, TNF-a, MDA, SAS, HAMD, AP, and BSS scores and increased SOD level (*p* < 0.05). The total effective rate was significantly increased (*p* < 0.05). The symptoms of angina attack frequency, nitroglycerin use, chest pain and tightness, shortness of breath, and emotional upset were also improved. DNJ reduced IKK and NF-κB levels and increased IkBα level (*p* < 0.05).

**Conclusion:** The DNJ in mulberry leaves improved the SAP of patients with CHD and BSS by increasing their antioxidant and anti-inflammatory capacities.

## Introduction

Cardiovascular disease is a potentially serious threat to public health and has hindered social and economic development (Joseph et al., 2017; Pasquel et al., 2018). Coronary heart disease (CHD) is a common cardiovascular disease and is a main cause of disability and death (Awerbach et al., 2018; Dong et al., 2018).

Angina pectoris (AP), which is a common symptom in patients with CHD and blood stasis syndrome (BSS), is difficult to treat due to the lack of effective drugs (Qian et al., 2013). Traditional Chinese medicine plays an important role in improving the symptoms and life quality of patients with CHD (Zhang et al., 2017; Chen et al., 2018b). The effectiveness of traditional Chinese medicine helps to develop personalized medicine (Wang et al., 2010). Chinese herbal medicine has been increasingly used to treat AP due to its effectiveness and safety with few side effects (Chen et al., 2018a). Herbs have been widely used in the prevention of cardiovascular diseases. Plants contain many phytochemicals that exert protective function by reducing the risk of various disorders (Al Disi et al., 2016). Certain herbs and spices can control blood pressure in hypertensive and pre-hypertensive patients (Driscoll et al., 2019). Botanicals have also been used to prevent atherosclerosis (Al-Shehabi et al., 2016). Danshen, the dried root of *Salvia miltiorrhiza*, can regulate lipoprotein metabolism, oxidation, and inflammation and protect vascular endothelia by affecting related signaling pathways. The results reflected the multi-component and multi-target characteristics of danshen and its therapeutic potential in heart diseases (Zhang et al., 2018).

1-Deoxynojirimycin (DNJ) is a unique polyhydroxy alkaloid that is the main active component in mulberry leaves (Jiang et al., 2014). DNJ is a competitive inhibitor of α-glucosidase, inhibiting the digestion and glucose absorption of disaccharides and thereby lowering blood glucose levels (Cai et al., 2017). DNJ is a potent α-glucosidase inhibitor with strong affinity toward α-glucosidase. DNJ can competitively inhibit the binding of maltoseucrose and other disaccharides to α-glucosidase and prevent the breakdown of disaccharides to form glucose. Thus, these disaccharides cannot be digested and absorbed and are passed into the large intestine and eventually excreted into feces. DNJ reduces glucose absorption and lowers blood sugar levels.

DNJ and its derivatives can effectively inhibit infection by HIV, HCV, and other viruses (Jacob et al., 2007; Onose et al., 2013). DNJ isolated from mulberry leaves exhibits strong inhibitory function on the oral pathogenic bacterium *Streptococcus* mutans (Hasan et al., 2014). DNJ protects against obesity-induced hepatic lipid abnormalities and mitochondrial dysfunction (Do et al., 2015). DNJ also promotes weight loss by increasing adiponectin levels, which play an important role in energy intake and in the prevention of diet-induced obesity. Further work showed that DNJ reduces obesity by moderating feeding behavior and endoplasmic reticulum stress in the central nervous system (Kim et al., 2017). DNJ may show protective effect against stable AP (SAP) in BSS patients. However, the effects of DNJ on SAP and the molecular mechanism involved have never been reported. Therefore, this study explored the effects of DNJ on SAP in patients with CHD and BSS.

## Materials and Methods

### DNJ Purification and HPLC Analysis

Mulberry (*Morus indica L.*, voucher number HDIEC-2016198) leaves were purchased from Huzhou Daybreak Import & Export, Co., Ltd. (Zhejiang, China) and deposited in the Herbarium of Nanjing University (Nanjing, China). Approximately 1 kg of dried mulberry leaf powder was dissolved in a 5 L round bottom flask, and 5 L of 0.1 M citrate buffer (pH 4.0) was added. The mixture was sonicated at 500 W for 30 min and filtered, and residues were discarded. The crude extract of mulberry leaves was concentrated and slowly added to a pretreated D72 type cation-exchange resin chromatography column. The resin was rinsed with ddH_2_O at a flow rate of 10 mL/min. DNJ was washed until the effluent solution was colorless then eluted with a 0.25 M aqueous ammonia solution at the same flow rate to collect the eluent at pH 9–12. The collected eluate was concentrated under reduced pressure to a volume, lyophilized, and stored for subsequent use. A total of 100 μg of the sample was accurately weighed and dissolved in ddH_2_O, and the volume was adjusted to 1 mL. The content and purity of DNJ were determined by HPLC pre-column derivatization.

A total of 10 mg of DNJ standard was dissolved in a 10 mL tube and diluted to 10 mL with distilled water to obtain a 1 mg/mL standard solution. The standard solutions were separately diluted to a standard solution series at concentrations of 5, 10, 20, 30, 40, and 50 μg/mL. A total of 10 μL of mulberry leaf extract was placed in a centrifuge tube and added with 10 μL of 0.4 M borate solution pH 8.5, followed by 20 μL of 5 mM FMOC-C1 acetonitrile solution. The solution was mixed and allowed to react in a 25°C water bath for 30 min. After the reaction, the mixture was added with 10 μL of 0.1 M glycine solution and unreacted derivatization reagent diluted to 1 mL with 1% acetic acid. The mixture was then stirred and filtered with a 0.45 μm filter. The following analysis conditions were used: column, Inertsil HPLC-NH2 analytical column (4.6 mm × 250 mm, 5 um); mobile phase, acetonitrile:water (75:25, v/v); flow rate, 1.0 mL/min; column temperature, 30°C; analysis time, 30 min; UV detector wavelength, 256 nm; and the injection volume, 10 μL.

### Patients

Before the experiments were performed, all protocols were approved by the Human Research Committee of the First Hospital of Jilin University (Approval No. 20161201DY). Written informed consents were obtained from the participants of this study. A total of 144 patients with SAP and BSS were recruited from December 2016 to September 2017. The patients were diagnosed according to the diagnosis guidelines for SAP (Messerli et al., 2006) and ischemic heart disease (IHD) (Shah, 2018). A history or at least one case of myocardial infarction was confirmed by coronary angiography. The degree of stenosis of coronary artery was >50%. The diagnostic criteria for BSS were based on the “Diagnostic Criteria for Coronary Heart Disease and Blood Stasis Syndrome” (Fu et al., 2012; Li et al., 2014).

### Inclusion Criteria

All patients satisfied the above criteria for SAP, BSS, and IHD. The patients were aged 35–80 years. The patients demonstrated SAP symptoms, including chest pain and tightness, shortness of breath, and emotional distress.

### Exclusion Criteria

Patients with the following cases were excluded from the study: SAP caused by other heart diseases; history of trauma, surgical infection, and fever; undergone or planned to receive coronary intervention in the past 1 month; severe heart failure with an ejection fraction (EF) of < 35%; malignant tumors, infections, blood diseases, mental illness, and other serious diseases; pregnancy or lactation; and serious complications that limit their participation in the experiment.

### Patient Grouping

A power test was used to calculate the population size with a power of 0.9 and an α of 0.5. The required population size was 140, with 70 patients in each group. A total of 144 patients were evenly divided into the control group (CG) and treatment group (EG) by using a computer-generated random number table. The patients in the CG and EG received conventional treatment and oral 10 mg of DNJ daily, respectively. The entire treatment duration was 4 weeks.

### Outcome Measure

The echocardiography of each patient was examined by using a GE Vivid E9 Doppler diagnosis system (GE Healthcare, Princeton, NJ, United States) with M5S-d and 4V probes, and the image was captured with an Echo PAC workstation. The left side of the person was examined in a calm position. The M5S-d probe was used to collect 2-D gray-scale dynamic images of the patient’s three cardiac cycles, including apical four-chamber heart, two-chamber heart, and left ventricular long axis. The 4V probe was placed on the apex position, and 3-D dynamic images were collected and analyzed. Echocardiographic analysis was performed using the following parameters: left atrial diameter, end-diastolic interventricular septal thickness, left ventricular end-diastolic diameter, left ventricular end-systolic diameter, left ventricular mass index (LVMI), left ventricular EF (LVEF), E′/A′ ratio (pulsed), left ventricular posterior wall thickness, and mean value of left ventricular outflow tract pressure gradient (LVOTPG, pulsed). Ascending aortic elasticity was evaluated by using the following indicators: aortic diastolic internal diameter (ADD) and aortic systolic inner diameter (ASD). The forward movement of the anterior wall of the ascending aorta was measured. The aortic elasticity index used was aortic distensibility. Aortic strain (AS) was calculated as follows: AS = 100 × [(ASD-ADD)/ADD], and aortic stiffness index was evaluated. The anterior wall systolic velocity of ascending aorta, early diastolic velocity, and late diastolic velocity were also measured. The patients recorded their walking distance in meters before experiencing angina during daily activity.

Changes in inflammatory factors and oxidative stress index, including high sensitivity C-reactive protein (hs-CRP, Cat. No. DEIA-BJ553, CD Creative-Diagnostics, Shirley, NY, United States), interleukin-6 (IL-6, Cat. No. ab47215, Abcam, Chicago, IL, United States), and tumor necrosis factor-α (TNF-α, Cat. No. ab181421), were compared before and after treatment by using corresponding ELISA kits. Superoxide dismutase (SOD) was measured by an automatic biochemical analyzer (Beckman Coulter, Inc., Brea, CA, United States). Malondialdehyde (MDA) was detected by a thiobarbituric acid reaction test (Baldi et al., 1993).

Zung’s Self-Rating Anxiety Scale (SAS) (Samakouri et al., 2012) and Hamilton Depression Scale (HAMD) (Vindbjerg et al., 2018) scores were compared before and after treatment. The improvement of AP and BSS scores was also measured. The symptoms associated with SAP, including chest pain and tightness, shortness of breath, emotional distress, and other clinical symptoms, were measured.

### Measurement of Therapeutic Effectiveness of SAP

Therapeutic effectiveness was defined as remarkably effective when the number of angina attacks or nitroglycerin decreased by >80%:, effective when the number of angina attacks or nitroglycerin decreased by 50 to 80%, Invalid when the angina was unchanged or worsened and when the number of angina attacks or nitroglycerin was reduced by <50%.

### Western Blot Analysis

Decreased transcriptional activity of the nuclear factor of transcription kappa B (NF-κB) is associated with the improvement of angina (Yang et al., 2010). The effects of DNJ on NF-κB pathway and related factors in SAP patients were investigated. Venous blood was collected on an empty stomach in the morning before and after treatment. The antibodies purchased included anti-IKKα (ab32041, Abcam, Chicago, IL, United States), anti-inhibitor of kappa B α (IkBα, Santa Cruz Biotechnology, Santa Cruz, CA, United States), anti-NF-κB p65 (Santa Cruz Biotechnology, Santa Cruz, CA, United States, 1:1000), and HRP goat anti-mouse (IgG) secondary antibody (Ab205719, Abcam, 1:2000). Blood cells were lysed by SDS/proteinase K, and proteins were separated by 12% SDS-PAGE. After PAGE, the proteins were transferred to PVDF membranes and sealed with 5% skim milk at room temperature for 2 h. Primary antibodies were added (1:1000), incubated overnight at 4°C, and washed with TBST (10 mM Tris-HCl [pH 8.0], 150 mM NaCl, and 0.05% Tween 20). Goat anti-mouse HRP secondary antibody was added and incubated for 2 h at room temperature on a shaker. After the membrane was washed, the bands were scanned with a GE AI600 imager, and the results were analyzed by the Gel-ProAnalyzer 4.0 software (Media Cybernetics, Bethesda, MD, United States).

### Statistical Method

SPSS19.0 was used for the analysis of data statistics. Count data were expressed as %, and the comparison between groups was performed by χ^2^ test. The measurement data were tested for normality and conformed to the normal distribution. Comparison between the two groups was performed by a paired-sample *t*-test. The difference was considered statistically significant if *p* < 0.05.

## Results

### DNJ Purification

**FIGURE 1:**
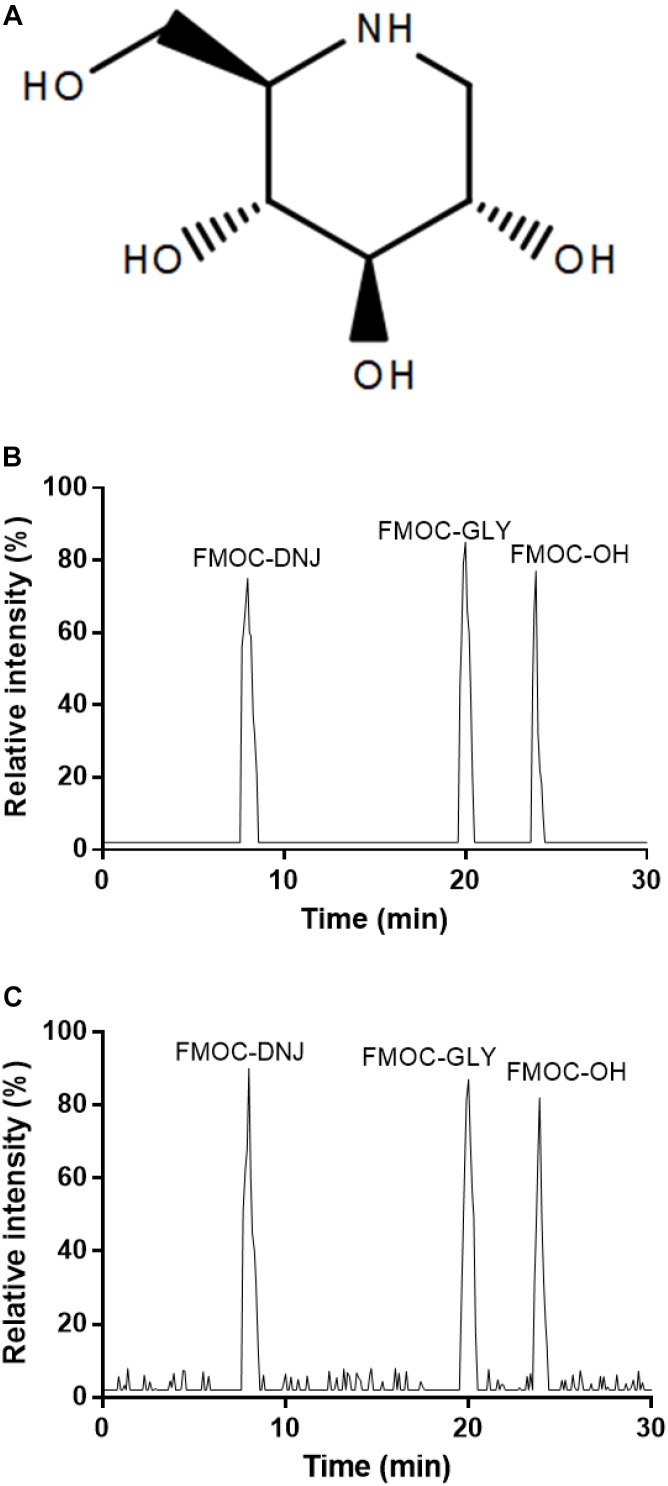
HPLC analysis of DNJ (1-deoxynojirimycin). **(A)** The structure of DNJ derivatives. **(B)** DNJ standard. **(C)** Purified DNJ derivatives from mulberry leaves.

DNJ is 1,5-dideoxy-1,5-imino-D-sorbitol (Figure 1A). After pre-column derivatization of DNJ standard and mulberry leaves, HPLC analysis was performed, and the results are shown in Figure 1B,C. Chromatographic peaks 1, 2, and 3 in the figure correspond to FMOC-DNJ, FMOC-GLY, and FMOC-OH, respectively. The DNJ in the mulberry leaf was well-separated from the adjacent components, and the derived reagent hydrolysates of FMOC-GLY and FMOC-OH did not interfere with measurement results.

### Clinical Characterization

A total of 144 eligible patients with CHD and BSS were enrolled, all of whom were diagnosed with SAP. After 4 weeks of therapy, 8 and 4 patients were withdrawn from the EG and CG, respectively. Statistical analysis was performed among 132 patients who completed the present experiment. The mean age of the two groups was 67.68 ± 8.09 years. No significant statistical difference in age, gender, and related risk factors for CHD was observed (*p* > 0.05, Table 1).

**Table 1:** Comparison of baseline clinical characters.

Parameters	Control group	Treatment group	*p*-values
Age (years)	68.16 ± 7.36	65.42 ± 8.48	0.15
Sex ratio (male/female)	42/26	44/20	0.552
Body mass index (kg/cm^2^)	25.47 ± 3.61	25.67 ± 3. 51	0.766
History of smoking (%)	18 (26.47)	28 (43.75)	0.141
Hypertension (%)	52 (76.47)	40 (62.5)	0.217
Diabetes (%)	28 (41.17)	20 (31.25)	0.402
Cholesterol (mM)	4.22 ± 1.10	4.39 ± 0.94	-0.495
Triglyceride (mM)	1.52 (1.12, 2.50)	1.99 (1.51, 2.34)	0.182
Low density lipoprotein (mM)	2.34 (1.78, 2.88)	2.31 (1.87, 2.74)	0.724
High density lipoprotein (mM)	1.19 (0.88, 1.42)	1.31 (1.13, 1.80)	0.095

*The statistical difference was significant if p < 0.05 vs. the control group.*

### DNJ Improved Conventional Echocardiographic Parameters

Before DNJ intervention, the statistical difference for all parameters was insignificant between the two groups (Table 2, *p* > 0.05). Compared with CG, EG showed increased LVEF and E′/A′ values and significantly reduced LVMI and LVOTPG after DNJ intervention (Table 2, *p* < 0.05). The other parameters were only slightly reduced.

**Table 2:** Conventional echocardiography.

Parameters	CG group	EG group
**Before treatment**		
Left atrial diameter/mm	41.80 ± 4.85	43.77 ± 4.94
Septal thickness/mm	17.13 ± 3.30	18.21 ± 3.86
Left ventricular end diastolic diameter/mm	43.51 ± 5.12	41.66 ± 4.71
Left ventricular end-systolic diameter/mm	28.15 ± 3.87	28.22 ± 3.86
Left ventricular posterior wall thickness/mm	10.65 ± 1.14	10.16 ± 0.81
Left ventricular ejection fraction/%	48.92 ± 7.95	49.51 ± 6.5
Left ventricular mass index/(g/m^2^)	139.26 ± 31.98	129.50 ± 28.57
E′/A′/(cm/s)	0.56 ± 0.13	0.60 ± 0.15
**After treatment**		
Left atrial diameter/mm	41.44 ± 13.8	38.81 ± 11.42
Septal thickness/mm	15.8 ± 9.13	13.39 ± 9.82
Left ventricular end diastolic diameter/mm	39.74 ± 13.13	36.01 ± 12.01
Left ventricular end-systolic diameter/mm	27.91 ± 11.6	25.76 ± 10.42
Left ventricular posterior wall thickness/mm	10.31 ± 3.06	9.73 ± 2.89
Left ventricular ejection fraction/%	51.2 ± 7.86	57.95 ± 6.96^*^
Left ventricular mass index/(g/m^2^)	134.79 ± 72.01	116.75 ± 51.65^*^
E′/A′/(cm/s)	0.58 ± 0.21	0.68 ± 0.28^*^
LVOTPG/mmHg	82.35 ± 37.19	70.06 ± 28.45^*^

*LVOTPG, the mean value of the left ventricular outflow tract pressure gradient. E′, peak early diastolic velocity of Tissue Doppler imaging (TDI) and A′, peak late diastolic velocity of TDI. ^∗^p < 0.05 vs. the CG group.*

### DNJ Improved Ascending Aortic Elasticity Parameters

Before DNJ intervention, the statistical difference for all parameters was insignificant between the two groups (Table 3, *p* > 0.05). After DNJ intervention, the aortic distensibility and atherosclerosis index in EG were lower than those in the CG (Table 3, *p* < 0.05). Early and late diastolic velocities in CG were also lower than those in EG (Table 3, *p* < 0.05).

**Table 3:** Ascending aortic elasticity.

Parameters	CG group	EG group
**Before treatment**		
The systolic velocity of the ascending aorta AWS′/(cm/s)	6.53 ± 2.05	6.01 ± 1.67
Early diastolic velocity, AWE′/(cm/s)	3.17 ± 0.85	3.24 ± 1.21
Late diastolic velocity, AWA′/(cm/s)	6.46 ± 2.07	6.17 ± 2.35
Aortic systolic inner diameter, ASD/mm	31.92 ± 5.41	31.58 ± 4.95
Aortic diastolic diameter, ADD/mm	30.72 ± 5.55	28.62 ± 3.91
Aortic distensibility, ADIS (cm^2^/dyne × 10^3^)	0.19 ± 0.09	0.18 ± 0.07
Atherosclerosis index, ASI	3.79 ± 0.41	3.70 ± 0.38
The degree of stenosis of coronary artery, %	63.12 ± 10.86	65.56 ± 9.37
**After treatment**		
The systolic velocity of the ascending aorta AWS′/(cm/s)	6.22 ± 5.92	5.37 ± 4.13
Early diastolic velocity, AWE′/(cm/s)	3.89 ± 2.11	4.75 ± 2.52^*^
Late diastolic velocity, AWA′/(cm/s)	6.21 ± 4.51	6.94 ± 3.03^*^
Aortic systolic inner diameter, ASD/mm	31.2 ± 11.02	29.48 ± 10.37
Aortic diastolic diameter, ADD/mm	30.53 ± 12.13	28.61 ± 8.01
Aortic distensibility, ADIS (cm^2^/dyne × 10^3^)	0.17 ± 0.05	0.13 ± 0.03^*^
Atherosclerosis index, ASI	3.69 ± 0.83	3.18 ± 0.69^*^
The degree of stenosis of coronary artery, %	72.56 ± 22.03	68.74 ± 18.89

*^∗^p < 0.05 vs. the CG group.*

### DNJ Intervention Increased Angina-Free Walking Distance

Before DNJ intervention, angina-free walking distance was insignificant between two groups (*p* > 0.05). After the 4-week treatment, DNJ intervention improved angina-free walking distance in EG (*p* < 0.05) but not in CG (*p* > 0.05, Table 4).

**Table 4:** The comparison of angina-free walking distance between two groups.

Groups	Before treatment	After treatment	*p*-values
CG	542.6 ± 55.1	555.2 ± 63.9	0.523
EG	539.5 ± 52.1	621.2 ± 67.8	0.026
*p*-Values	0.632	0.021	

*The statistical difference was significant if p < 0.05 vs. the control group.*

### DNJ Treatment Increased Anti-inflammatory Properties

Before the treatment, the statistical difference for the serum levels of hs-CRP, IL-6, and TNF-a was insignificant between the two groups (*p* > 0.05, Table 5). After the 4-week treatment, the serum levels of inflammatory factors hs-CRP, IL-6, and TNF-a in EG were reduced compared with those in CG (*p* < 0.05, Table 5). The results suggest that DNJ treatment increased the anti-inflammatory features of the patients.

**Table 5:** The comparison of serum levels of inflammatory cytokines before and after treatment.

Cytokines		Control group	Treatment group	*p*-values
CRP (μg/mL)	Before	4.65 ± 0.86	4.79 ± 0.81	0.501
	After	3.96 ± 0.83	3.57 ± 0.75	0.042
IL-6 (pg/mL)	Before	150.12 ± 17.04	150.68 ± 16.26	0.892
	After	146.95 ± 14.27	139.65 ± 14.64	0.048
TNF (pg/mL)	Before	66.98 ± 7.08	66.29 ± 7.17	0.692
	After	65.34 ± 6.35	62.11 ± 6.63	0.047

*The statistical difference was significant if p < 0.05 vs. the control group.*

### DNJ Treatment Increased Anti-antioxidant Activities

**FIGURE 2:**
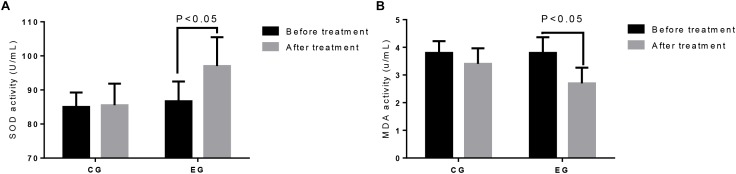
The comparison of antioxidant activities between two groups. **(A)** SOD activity. **(B)** MAD activity. The statistical difference was significant if *p* < 0.05 vs. the control group.

Before the treatment, the statistical difference for the serum levels of SOD (Figure 2A) and MAD (Figure 2B) was insignificant between the two groups (*p* > 0.05). After the 4-week treatment, the serum SOD levels (Figure 2A) were increased, whereas the MAD levels (Figure 2B) in EG were reduced compared with those in CG (*p* < 0.05). The results suggest that DNJ treatment increased the antioxidant capacities of the patients.

### DNJ Treatment Improved the Anxiety and Depression of Patients With SAP

Before the treatment, the statistical difference for the scores of SAS and HAMD was insignificant between the two groups (*p* > 0.05, Table 6). After the 4-week treatment, the SAS and HAMD scores in EG were reduced relative to those in CG (*p* < 0.05, Table 6). The results suggest that DNJ treatment improved the anxiety and depression of patients with SAP.

**Table 6:** The comparison of the scores of anxiety and depression between two groups.

Parameters		Control group	Treatment group	*p*-values
SAS	Before	51.18 ± 7.06	53.72 ± 9.73	0.227
	After	44.03 ± 6.92	47.56 ± 6.96	0.043
HAMD	Before	15.21 ± 6.25	14.50 ± 5.30	0.623
	After	13.06 ± 7.27	9.16 ± 5.15	0.014

*SAS, Self-Rating Anxiety Scale. HAMD, Hamilton Depression Scale. The statistical difference was significant if p < 0.05 vs. the control group.*

### DNJ Treatment Improved Therapeutic Results

After the 4-week treatment, the total effective rate in the EG (81.25%) was higher than that in the CG (47.06%, *p* < 0.05, Table 7). The findings suggest that DNJ treatment improved therapeutic results.

**Table 7:** The comparison of therapeutic effectiveness between two groups [Cases (%)].

Groups	Cases	Significant effective	Effective	Invalid	Total effective	*p*-value
Control group	68	0	32 (47.06)	36 (52.94)	32 (47.06)	0.005
Treatment group	64	14 (21.88)	38 (59.37)	12 (18.75)	52 (81.25)	

*The statistical difference was significant if p < 0.05 vs. the control group.*

### DNJ Treatment Improved SAP, BSS, and GIN Use

Before the treatment, the statistical difference for SAP scores, SAP frequency, BSS scores, and GIN use was insignificant between the two groups (*p* > 0.05, Table 8). After the 4-week treatment, SAP scores, SAP frequency, BSS scores, and GIN use were reduced in EG relative to those in CG (*p* < 0.05, Table 8). The results suggest that DNJ treatment improved SAP, BSS, and GIN use in patients with SAP.

**Table 8:** The comparison of the outcome of SAP, BSS, and GIN use between two groups.

Parameters		Control group	Treatment group	*p*-values
SAP scores	Before	12 (10, 14)	12 (10, 16)	0.356
	After	10 (8, 15)	6 (6, 8)	<0.01
SAP frequency	Before	4 (2, 4)	4 (2, 4)	0.879
	After	2 (2, 4)	2 (1, 3)	0.001
SAP duration	Before	3 (2, 4)	4 (2, 4)	0.711
	After	2 (2, 4)	2 (2, 2)	0.003
BSS scores	Before	25 (19, 27)	25 (17.25, 28)	0.892
	After	24 (18.5, 25)	17 (9.5, 17.75)	<0.01
GIN tablets daily	Before	4 (2, 4)	4 (2, 4)	0.132
	After	4 (2, 5)	2 (0.5, 2)	0.017

*SAP, stable angina pectoris; BSS, blood stasis syndrome. GIN, glyceryl trinitrate; the statistical difference was significant if *p* < 0.05 vs. the control group.*

### DNJ Treatment Improved SAP Symptoms

Before the treatment, the statistical difference for SAP symptoms was insignificant (Table 9, *p* < 0.05). After the 4-week treatment, the SAP symptoms, including chest pain, chest tightness, shortness of breath, and upset feeling, were evidently improved in the EG (Table 9, *p* < 0.05). The results suggest that DNJ treatment enhanced SAP symptoms.

**Table 9:** The comparison of SAP symptoms between two groups.

Parameters	Time	Control group	Treatment group	*p*-values
Chest pain	Before	3 (3, 6)	12 (6, 12)	0.296
	After	6 (6, 12)	6 (6, 6)	0.022
Chest tightness	Before	6 (6, 6)	6 (6, 12)	0.559
	After	6 (6, 12)	6 (6, 6)	0.004
Shortness of breath	Before	6 (6, 6)	4 (4, 8)	0.154
	After	6 (4, 8)	4 (1, 4)	0.003
Tired	Before	4 (4, 4)	6 (4, 8)	0.594
	After	4 (4, 8)	4 (4, 4)	0.061
Palpitate	Before	4 (4, 4)	4 (2, 4)	0.194
	After	2 (2, 4)	4 (2, 2)	0.063
Bitter	Before	2 (2, 2)	4 (2, 4)	0.358
	After	2 (2, 4)	2 (2, 2)	0.132
Upset	Before	2 (2, 2)	4 (2, 4)	0.407
	After	3 (2, 4)	2 (2, 2)	0.015
Total score	Before	19.41 ± 4.84	20.32 ± 4.54	0.392
	After	14.65 ± 2.65	11.22 ± 2.69	<0.01

*The statistical difference was significant if p < 0.05 the control group.*

### DNJ Increased IkBα Level and Reduced IKK and NF-κB Levels

**FIGURE 3:**
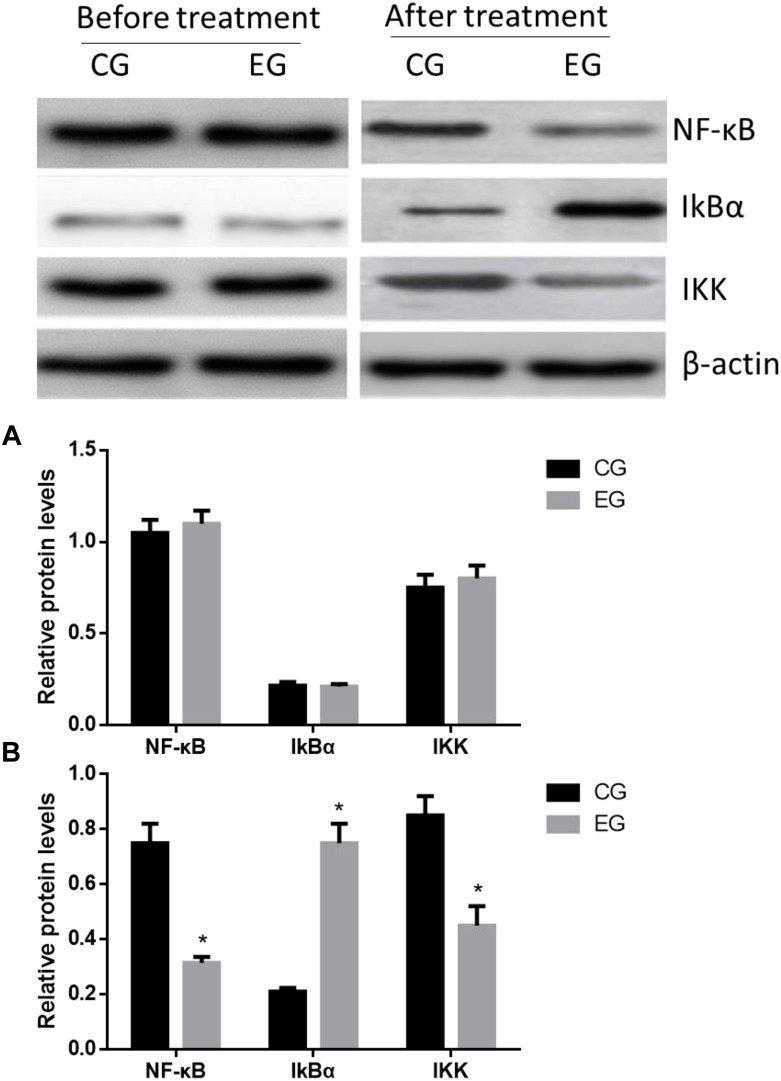
Western Blot analysis of relative protein levels between two groups. **(A)** Before treatment. **(B)** After treatment. ^∗^*p* < 0.05 vs. the CG group.

The statistical difference for the levels of IkBα, IKK, and NF-κB was insignificant between the two groups before treatment (Figure 3A, *p* > 0.05). The levels of IKK and NF-κB in the EG were significantly lower than those in the CG, whereas the IkBα level showed reverse results (Figure 3B, *p* < 0.05). These results suggest that DNJ reduced IKK and NF-κB levels and increased IkBα level.

## Discussion

Stable angina pectoris has become a global public health burden, and complementary treatment is often considered for patients with this disease. The key to complementary treatment is to find potential drugs for patients. As the main syndrome of CHD, SAP was reduced by DNJ treatment by affecting some parameters involved with conventional echocardiography and ascending aortic elasticity and by increasing angina-free walking distance. The present study showed that DNJ is a potential drug for SAP therapy in CHD patients with BSS.

Oxidative stress (Yan et al., 2009) and inflammatory responses (Militaru et al., 2013; Galon et al., 2015) are associated with SAP risks, whereas oxidative stress and inflammation can interact and promote each other (Herman et al., 2018; Zahran and Emam, 2018), possibly aggravating SAP. Oxidative stress and inflammation are associated with endothelial dysfunction (Zhenyukh et al., 2018), and impaired endothelial function results in blood flow decrease (Brunnekreef et al., 2012), vasospasm (Ganz et al., 1991), and embolus formation (Muci-Mendoza et al., 1980). Hs-CRP as a downstream inflammatory marker (Tayefi et al., 2017) and TNF-a and IL-6 (Zhou et al., 2013) as upstream inflammatory mediators are significant predictors of cardiovascular risk. The present study demonstrated that DNJ treatment reduced inflammatory responses (Table 5) and increased antioxidant activities (Table 6), contributing to the improvement of SAP symptoms.

Anxiety and depression are associated with the mortality of CHD and SAP patients (de Jager et al., 2018). The present findings suggested that DNJ treatment could ameliorate SAP by reducing SAS and HAMD scores and GIN use (Table 6). In clinical treatment, SAP symptoms could be alleviated by improving the anxiety and depression of patients (Table 8). The therapeutic results were improved, and SAP symptoms were reduced after treatment (Table 9). The total therapeutic results in EG were better than those in CG. DNJ treatment showed good clinical effect on the anxiety and depression of patients with SAP and BSS.

Exploring an effective therapeutic method of SAP is very important in its prevention. NF-κB participates in various physiological functions, such as inflammation, immune response, and apoptosis, by regulating the expression of various genes associated with AP (Ritchie, 1998), whereas IKBα is a key inhibitor of the NF-κB pathway. The present results showed that DNJ inactivated the NF-κB pathway by increasing IkBα levels.

The present work suffered from some limitations. A small population size of 132 patients with SAP, CHD, and BSS were enrolled in this study. This study mainly focused on the anti-inflammatory and anti-oxidative properties and anxiety and depression of patients with SAP. The effects of DNJ on other symptoms of SAP were not investigated. Further work must confirm the present conclusion in a large population.

## Conclusion

DNJ intervention improved some parameters involved with conventional echocardiography and ascending aortic elasticity and increased angina-free walking distance. DNJ treatment reduced the serum levels of hs-CRP, IL-6, TNF-a, SOD, and MDA in the patients with SAP and BSS. Moreover, DNJ demonstrated anti-inflammatory and antioxidant properties. DNJ reduced SAS and HAMD scores in CHD patients, reflecting that DNJ regulated anxiety and depression in patients with SAP. DNJ treatment also decreased the SAP and BSS scores, suggesting that DNJ improved the curative effects in patients with SAP. Furthermore, DNJ improved the symptoms of chest pain, chest tightness, shortness of breath, emotional upset, and other clinical symptoms, all of which are associated with SAP.

## Ethics Statement

All procedures were approved by the human research ethical committee of The First Hospital of Jilin University (Changchun, China).

## Author Contributions

YM and SY conceived and designed the experiments and wrote the manuscript. WL and YG contributed to the evaluation of the results and corrected the manuscript.

## Conflict of Interest Statement

The authors declare that the research was conducted in the absence of any commercial or financial relationships that could be construed as a potential conflict of interest.
